# Engineering challenges in microphysiological systems

**DOI:** 10.4155/fsoa-2017-0049

**Published:** 2017-06-09

**Authors:** Yu Shrike Zhang

**Affiliations:** 1 Division of Engineering in Medicine, Department of Medicine, Brigham and Women's Hospital, Harvard Medical School, Cambridge, MA 02139, USA

**Keywords:** bioanalysis, biofabrication, blood surrogate, microfluidics, microphysiological systems, organ-on-a-chip, personalized medicine, scaling

“Microphysiological systems are emerging models of human physiology with broad applications in biomedicine, which are yet associated with several major engineering challenges that need to be addressed”.

## Modeling human physiology with the microphysiological systems

In humans, tissues and organs are made of hierarchically assembled structures of multiple compositions to achieve biological functions. The different tissues and organs are then organized in a specific order enabled by a circuitry of vascular network, further achieving physiological interactions. On the one hand, many of these complex elements cannot be readily reproduced on the conventional planar, static cell culture systems already used in biology and medicine for over a century [1]. On the other hand, while preclinical animal models are both biologically and physiologically capable, their relevancy to the human system remains questionable, often leading to inaccurate clinical translation of assay results [2].

To this end, microphysiological systems, which are miniaturized biomimetic *in vitro* human tissue and organ models built from a combination of biological and engineering approaches, usually feature much higher performance in recapitulating the functions of their *in vivo* counterparts [3–7]. A microphysiological system usually consists of three main aspects: the organoid that is biologically relevant, often generated through principles including developmental biology [8], tissue engineering [9], bioreactor designs [10,11] and their combinations; the biophysical cues that represent the local niches, such as the supporting matrix with tissue-matching properties [12], shear stress/interstitial pressure [13] and biomechanical strains [14]; and the circulatory system that brings in physiological relevance, to enable communications among different organoids [5].

The ability to interconnect multiple organoids together in a fashion so that the integral platforms reproduce their desired human physiology, forms the unique strength of the microphysiological systems. This is because of the fact that in the human body no single tissue or organ is isolated, where the function and behavior of one is closely dependent on the others through biophysical and biochemical interactions. Sung and Shuler piloted the micro cell culture analog devices where up to ten types of organs were integrated to study their interactions [15]. Wikswo proposed the microphysiological systems with built-in valves to control the ‘blood flow’ among different organs [4]. More recently, a platform was developed to support long-term coculture of multiple human organoids [16], and a portable, reconfigurable multi-organ system with onboard microfluidic flow control has been reported [17]. Further development of these biomimetic microphysiological systems are anticipated to enable the construction of viable ‘human-on-a-chip’ platforms, which together with the advances in stem cell technologies could make personalized precision medicine possible. However, several major engineering challenges remain along the journey to achieve this ambitious goal.

## Biofabrication: making better organoids

Generating functional organoids typically requires deep biology, including those either derived from mature tissues or differentiated from stem cells with refined protocols [18,19]. Since the late 2000s, biologists have grown a wide variety of near-physiological organoids, such as cerebral cortex, intestine, optical cup, pituitary gland, kidney, liver, pancreas, neural tube, stomach, prostate, breast, heart and lung [19]. While they behave similarly to their *in vivo* counterparts, the processes of organoid generation based on principles of developmental biology and cell self-organization are usually lengthy at relatively low throughputs. On the other hand, tissue engineers have been able to adapt techniques used in engineering functional tissues previously aimed for regeneration purposes to also build miniaturized tissue models [20], which are often faster and more convenient than biological methods, although the generated tissues are in general less functional. It is perhaps the convergence of these two complementary approaches combining biology with engineering techniques (e.g., micro-/nanotopography, photolithography and bioprinting) to impart biomimetic architectures that would eventually benefit the faithful production of human organoids at shorter turnover times and higher throughputs.

## Blood surrogate: keeping them all functional

To ensure proper functions of a microphysiological system, a common medium, termed the blood surrogate, must be used to perfuse its entire circulation in an effort to maintain the system's homeostasis by transporting nutrient, oxygen and waste molecules to and from each organoid, and support the functionality of each organoid [4,21]. The blood surrogates can be developed by carefully tuning the compositions of key components in the culture medium for each individual cell type, which is nontrivial, considering that every organoid is different in its cell types, every microphysiological system is distinctive in its organoid types and every configuration of the microphysiological system is unique. The complexity for such developments exponentially expands as the number of organoids to be connected in a microphysiological system is increased, further necessitating meticulous optimization processes. In addition, while most cell culture media contain serum, future advances in the blood surrogate are progressively trending toward the use of additives of well-defined compositions to improve the standardization of these formulations.

## Scaling: ensuring the right proportions

Scaling is another critical consideration in the design of microphysiological systems [21,22]. This can be divided into two closely related levels: the scaling between the organoids and their human counterparts and each other, which determines whether the microphysiological systems could accurately model the basic human physiology [21], as well as the scaling of the biophysical parameters including, for example, the compartmentalization of the organoids in a fluidic circuitry, the flow parameters and the intake/excretion pathways, where pharmacokinetics and pharmacodynamics of molecules may be simulated [16,22]. To date, several direct and allometric scaling laws have been proposed such as those by organ weight, volume and blood flow [21]. More recently, a multifunctional scaling approach was further introduced to design microphysiological systems based on mechanistic modeling of the biological mechanisms, specification of an objective function and identification of design parameters [23], which in comparison, could be more precisely tuned toward each specific application.

## Bioanalysis: monitoring the responses

The microphysiological systems are self-contained, similar to the human body. Under this scenario, while it is still necessary to conduct ‘biopsies’ as the (minimally) invasive or endpoint assays, a more intuitive approach is to sample the ‘blood’ *in situ* for long-term analysis of the responses of the organoids, the same way how the patients are examined for labs in the hospital. The most convenient approach falls to the design of a sampling outlet that can be activated to sample certain amounts of medium at predetermined time intervals for off-chip assessments such as the conventional enzyme-linked immunosorbent assay (ELISA) and mass spectrometry (MS). Alternatively, it would make the procedures more physiologically relevant by integrating on-board biochemical sensors with the microphysiological systems for monitoring the analyte levels produced by the organoids, to reduce labor and variations due to human errors [24]. Physical sensing units should further be included to allow for precise assessment and reverse modulation of physical microenvironments in which the organoids reside [25]. The capability to introduce various imaging abilities that enable noninvasive and *in situ* characterization of the organoids is of equal importance to understand their behaviors [24].

## Conclusion

Microphysiological systems have emerged as enabling platform technologies for modeling human physiology, featuring structural and functional analogy to ensure biological relevance as well as physiological simulation through compartmentalized microfluidic interconnections. As such, they are potentially much more realistic than the conventional planar, static cell culture models and closer to the human system than animals. It is anticipated that, by further overcoming several major challenges associated with building the microphysiological systems, they will eventually find widespread biomedical applications across a variety of fields spanning from basic cell biology to drug development.
